# Posterior instrumentation after a failed balloon kyphoplasty in the thoracolumbar junction: a case report

**DOI:** 10.1186/1752-1947-8-189

**Published:** 2014-06-13

**Authors:** David Cumming, Thomas Pagonis, Ryan Wood

**Affiliations:** 1 Trauma & Orthopaedic Department, Spinal Unit, The Ipswich Hospital, Heath Road, Ipswich IP4 5PD, UK

**Keywords:** Balloon kyphoplasty, Lumbar fracture, Osteopenia, Osteoporosis, Vertebral fracture complications

## Abstract

**Introduction:**

Balloon kyphoplasty provides symptomatic relief of vertebral compression fractures in elderly patients. Peri-operative complications are rare; however, they can potentially be devastating. To the best of our knowledge, complications during balloon kyphoplasty have not been described previously in published case reports.

**Case presentation:**

A 66-year-old man who was a farmer of Caucasian origin presented with a 6-month history of back pain after a fall. We discovered a significant T12 wedge compression fracture, so we performed a T12 balloon kyphoplasty. Approximately 2 weeks after being discharged from our hospital, the patient presented with increasing back pain. He presented for a second time with excruciating pain on the left side of his thoracolumbar region, so he was admitted to our ward. X-rays did not show any further fractures or compromise, but magnetic resonance imaging showed extensive edema in the T11 and L1 vertebral bodies as well as fluid tracking from the T11-T12 disc into the vertebral body. Nine days after being discharged, the patient presented to the outpatient clinic with severe back pain. Magnetic resonance imaging at that visit showed edema at the levels above and below the T11/T12 disc. He was put into a brace and given 300mg of morphine, which did not provide any pain resolution. Posterior instrumentation from T9 to L2 (pedicle fixation of T9-T10 as well as L1-L2, rods in between and a crosslink above T11-T12) was performed as the final treatment, and the patient was discharged uneventfully.

**Conclusion:**

Patients presenting with residual pain over a previous balloon kyphoplasty level should raise high suspicion for a fracture or complication involving the levels above and/or below the balloon kyphoplasty. The best way to treat fractures that develop after a failed balloon kyphoplasty is to instrument and fuse posteriorly. Our present case report shows that a high level of suspicion for possible new fractures should be maintained for all similar cases.

## Introduction

Balloon kyphoplasty (BKP) has been shown to provide symptomatic relief of vertebral compression fractures in elderly patients refractory to conservative medical therapy [1-3]. Brace treatment and open surgical intervention are less desirable treatments in this population because of the associated medical comorbidities. As such, BKP has been advocated as a minimally invasive treatment option for symptomatic compression fractures. BKP involves the inflation of a balloon to create a cavity and restore vertebral height. This procedure is followed by injection of cement into the fractured vertebra. Peri-operative complications related to the treatment of vertebral compression fractures are rare; however, when they occur, they can potentially be devastating [4-7]. The use of cement extravasation has been reported. Other procedural complications of vertebral augmentation that have been described include fractured transverse processes or ribs, dural tears, discitis and subcutaneous hematomas. In general, complications during BKP have been published in case reports [8,9].

## Case presentation

**Figure 1:**
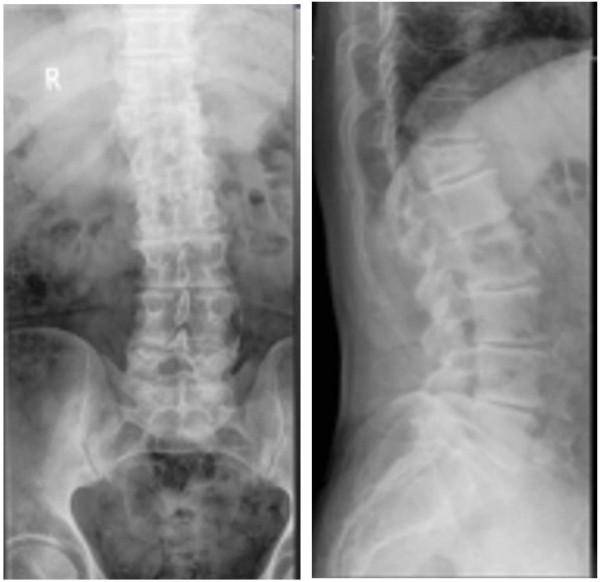
Plain radiographs obtained at baseline showing the patient’s T12 fracture in standing anteroposterior and lateral views of the lower thoracic and lumbar spine.

**Figure 2:**
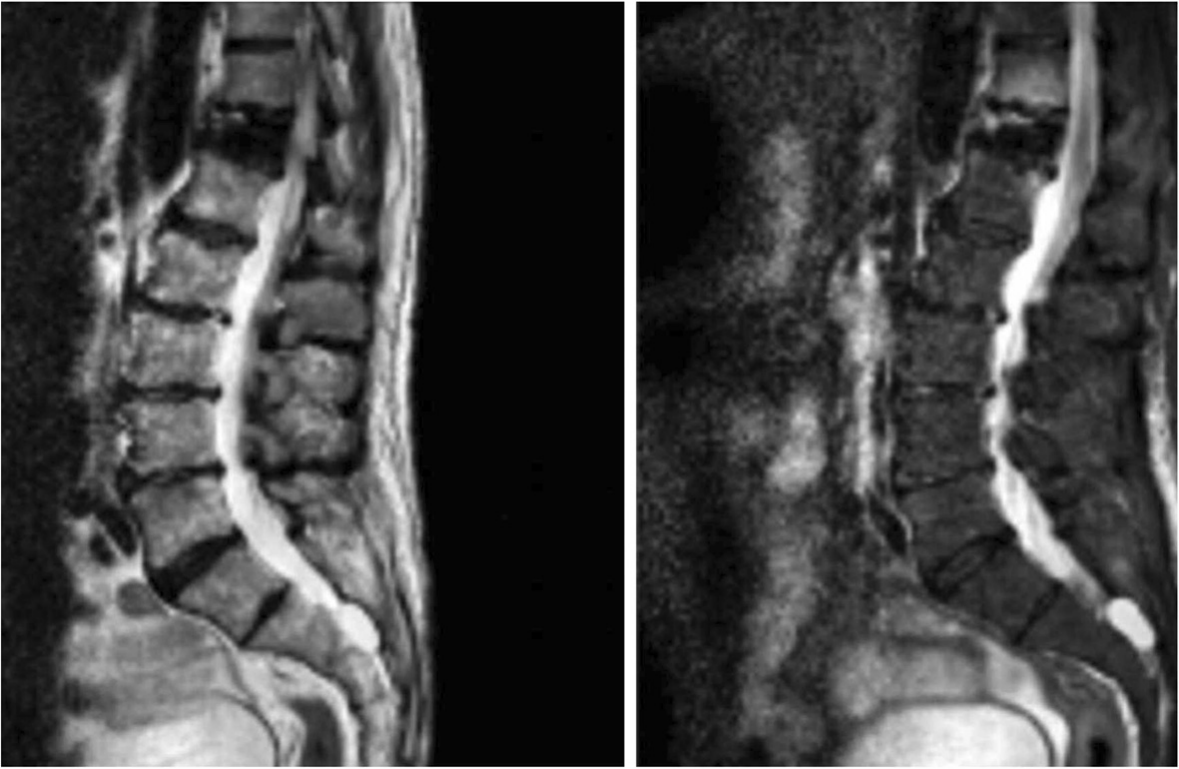
T2-weighted magnetic resonance imaging scans and short tau inversion recovery sequence sagittal cuts showing wedge fracture of T12.

**Figure 3:**
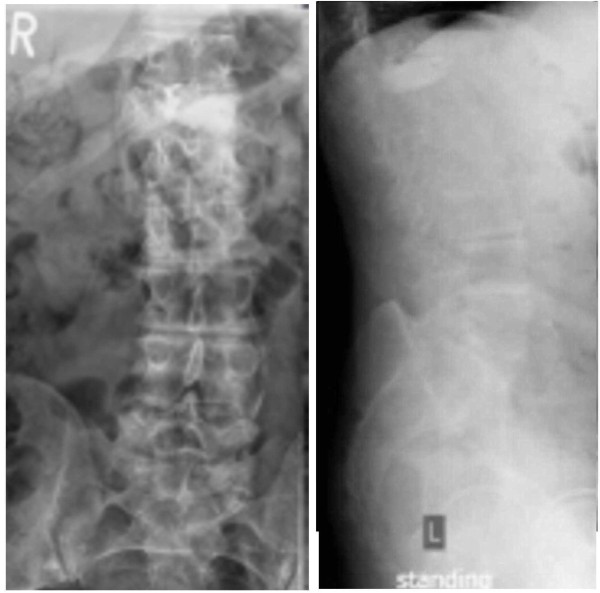
Post-kyphoplasty X-rays show anteroposterior and lateral views of the lumbar spine.

**Figure 4:**
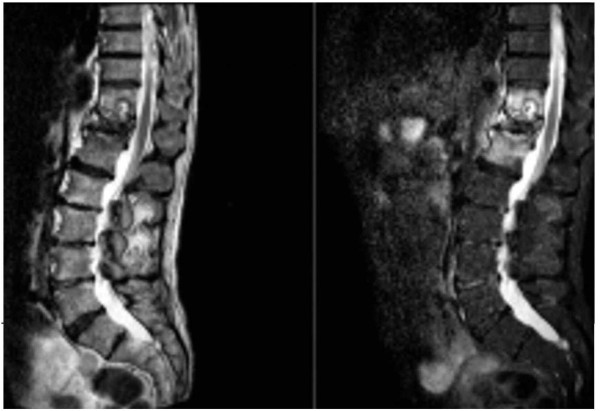
T2-weighted magnetic resonance imaging scan and short tau inversion recovery sequence sagittal cuts showing further effusion of T11.

A 66-year-old man who was a farmer of Caucasian origin presented to our specialist clinic after being referred by his general practitioner. He had a 6-month history of back pain in the thoracolumbar region, which was more pronounced when he stood up for a long time and for which he required regular analgesia. He did not state any bladder or bowel disturbance and had no other neurological disturbances. He stated that 6 months previously he had fallen approximately 10 feet from a combine harvester and immediately developed back pain. The patient was a non-smoker and a social drinker of alcohol, and his past medical history included myocardial infarction, deep vein thrombosis/pulmonary embolism, hay fever, asthma, emphysema, diabetes and under-active thyroid. He was taking thyroxine, paracetamol and morphine. A dual-energy X-ray absorptiometry scan demonstrated no evidence of osteoporosis or osteopenia. His clinical examination demonstrated tenderness over T12 but normal distal neurology with normal reflexes and no clonus. Radiographs showed a significant T12 wedge compression fracture (Figure 1). He was referred for magnetic resonance imaging (MRI) (Figure 2) on the basis that he might be a good candidate for kyphoplasty. The MRI scan showed edema within the body of T12 on the short tau inversion recovery sequence. Blood samples taken upon admission did not reveal any abnormality.Two months after the initial consultation, we performed a T12 kyphoplasty with no complications (Figure 3).Approximately 2 weeks after being discharged, the patient presented to the emergency department of our hospital with increasing back pain that improved at rest and with significant amounts of pain medication. He presented for a second time to the emergency department with excruciating pain on the left side of his thoracolumbar region, so he was admitted to our ward. X-rays did not show any further fractures or compromise, but MRI (Figure 4) showed extensive edema in the T11 and L1 vertebral bodies with fluid tracking from the T11-T12 disc into the vertebral body, which was a strong indication of possible pre-disposition to further osteoporosis involvement. All blood tests performed at this time, including full blood count (FBC), C-reactive protein (CRP) and erythrocyte sedimentation rate (ESR), were normal. The patient was discharged 9 days later after receiving facet joint block injections (1ml of 40mg kenalog + 1ml of Marcaine® 0.25%).

**Figure 5:**
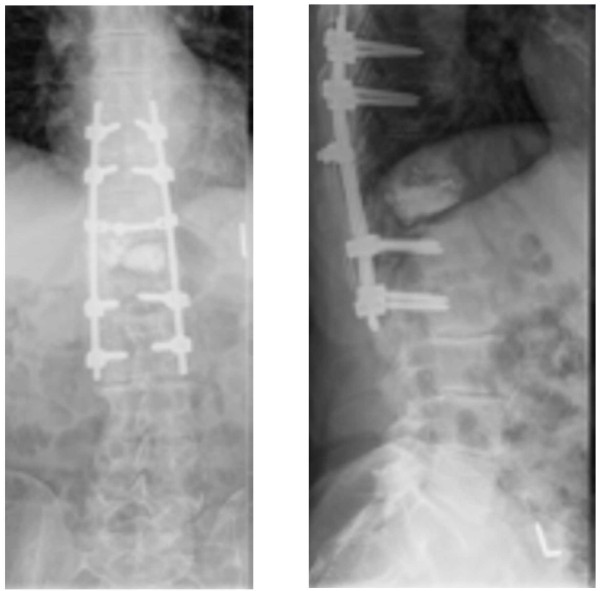
Post-operative X-rays of the patient’s lower thoracic and lumbar spine (anteroposterior and lateral views) showing posterior fixation.

The patient presented to the outpatient clinic of our hospital 9 days later with severe back pain. He stated that he had experienced no relief from the facet joint block injections. A MRI study showed edema on the level above and below the facet joint block injection site at the T11/12 Facet joints. He was put into a brace and blood samples were collected for FBC, urea and electrolytes, CRP and ESR. The only abnormal value was CRP (10mm/h), so the patient was put on 300mg of morphine, which did not lead to pain resolution. At the multi-disciplinary team meeting on the same day, the general consensus was that the patient should undergo a posterior fixation of two levels above and below the fracture site (T12), with a biopsy taken at the same time.Posterior instrumentation from T9 to L2 (pedicle fixation of T9-T10 as well as L1-L2, rods in between and a crosslink above T11-T12) was performed 1 month after the patient’s last admission (Figure 5), and he was discharged uneventfully 5 days after that. A biopsy was taken during the instrumentation procedure.

Twenty days after the procedure the patient was re-reviewed and found to be pain-free while his pain medication had been reduced. The results of the biopsy showed a possible diagnosis of osteoporosis, but nothing else of note. The patient was reviewed 3 months after surgery, at which time his condition had improved significantly.

## Discussion

Complications of BKP are usually poorly reported. In general, vertebral compression fractures occur in elderly patients with multiple medical comorbidities. The reporting of medical complications may be subject to bias because BKP is often an outpatient procedure and thus complications may not be reported during the hospitalization. We wish to emphasize the poor overall condition of patients who typically experience compression fractures, whether osteoporotic or pathologic. The incidence of procedure-related complications appears to be higher for vertebroplasty (VP) than for BKP in all studies and prospective studies. This trend may be explained in part by historical context. VP was developed before BKP. The two procedures share the same approach, but complications encountered in earlier VP procedures may not be encountered in BKP because of the increased technical experience gained over time. VP is associated with a higher rate of cement leakage, both symptomatic and asymptomatic, in patients with osteoporotic and/or pathologic conditions. Furthermore, VP in pathologic fractures is associated with a higher cement leak rate than VP in osteoporotic fractures. It appears that VP may be associated with an increased new fracture rate compared to BKP. This result should be interpreted cautiously because the occurrence of new fractures at previously unaffected spine levels may be multi-factorial. Variability in fracture reporting can confound these results because only symptomatic fractures are likely to be reported.

## Conclusion

Patients presenting with residual spinal pain over a previous BKP site should raise high suspicion for a fracture or complication involving the levels above and/or below the VP site. The best way to treat fractures that develop after a failed VP is to perform instrumentation and fusion posteriorly.

## Consent

Written informed consent was obtained from the patient for publication of this case report and any accompanying images. A copy of the written consent is available for review by the Editor-in-Chief of this journal.

## Competing interests

The authors declare that they have no competing interests.

## Authors’ contributions

DC was the operating surgeon and treating physician, treated the patient and was a major contributor to the writing of the manuscript. TP and RW analyzed and interpreted the patient data and were major contributors to the writing of the manuscript. All authors read and approved the final manuscript.
